# Lung Epithelial TRPA1 Mediates Lipopolysaccharide-Induced Lung Inflammation in Bronchial Epithelial Cells and Mice

**DOI:** 10.3389/fphys.2020.596314

**Published:** 2020-11-17

**Authors:** Hsin-Kuo Ko, An-Hsuan Lin, Diahn-Warng Perng, Tzong-Shyuan Lee, Yu Ru Kou

**Affiliations:** ^1^Department of Chest Medicine, Taipei Veterans General Hospital, Taipei, Taiwan; ^2^School of Medicine, National Yang-Ming University, Taipei, Taiwan; ^3^Department of Physiology, School of Medicine, National Yang-Ming University, Taipei, Taiwan; ^4^Department of Physiology, University of Kentucky, Lexington, KY, United States; ^5^Graduate Institute and Department of Physiology, College of Medicine, National Taiwan University, Taipei, Taiwan

**Keywords:** lipopolysaccharide, TRPA1, calcium, reactive oxygen species, signaling pathway, lung epithelial cell, lung inflammation

## Abstract

Toll-like receptor (TLR) 4 was originally thought to be the sole pattern recognition receptor for lipopolysaccharide (LPS). Transient receptor potential ankyrin 1 (TRPA1), a Ca^2+^-permeant channel, has been suggested as a non-TLR receptor membrane-bound sensor of LPS. We recently reported that TRPA1 is expressed in lung epithelial cells (LECs) and mediates lung inflammation induced by cigarette smoke. However, the role of TRPA1 in LPS-induced lung inflammation has not been conclusively defined, and its underlying cellular mechanisms remain unclear. In this study, our *in vitro* results showed that LPS sequentially produced a cascade of events, including the elevation of intracellular Ca^2+^, the activation of NADPH oxidase, increase in intracellular reactive oxygen species (ROS), the activation of mitogen-activated protein kinase (MAPK)/nuclear factor-kB (NF-κB) signaling, and the induction of IL-8. The increase in intracellular Ca^2+^ was inhibited by HC030031 (a TRPA1 antagonist) but was unaffected by TAK-242 (a TLR-4 inhibitor). The activation of NADPH oxidase was prevented by its inhibitor apocynin, EGTA (an extracellular Ca^2+^ chelator), and HC030031. The increase in intracellular ROS was attenuated by apocynin, N-acetyl-cysteine (NAC, a ROS scavenger), EGTA, and HC030031. The activation of the MAPK/NF-κB signaling was halted by NAC, EGTA, and HC030031. IL-8 induction was suppressed by HC030031 and TRPA1 siRNA, and further reduced by the combination of HC030031 and TAK-242. Our *in vivo* studies showed that *trpa1^–/–^* mice exhibited a reduced level of LPS-induced lung inflammation compared with wild-type mice as evidenced by the alleviations of increases in vascular permeability, inflammatory cell infiltration, inflammatory cytokine levels, oxidative stress, and MAPK signaling activation. Thus, in LECs, LPS may activate TRPA1 resulting in an increase in Ca^2+^ influx. The increased intracellular Ca^2+^ leads to NADPH oxidase activation, which causes an increase in intracellular ROS. The intracellular ROS activates the MAPK/NF-κB signaling resulting in IL-8 induction. This mechanism may possibly be at work to induce lung inflammation in mice.

## Introduction

Sepsis is a systemic inflammatory state manifested by a complex interaction between infective microorganisms and host immune responses (Cecconi et al., 2018). Bacterial lipopolysaccharide (LPS), a constituent of the outer membrane of Gram-negative bacteria, is a potent inducer of pro-inflammatory activity (Neyen and Lemaitre, 2016). The lung epithelium is positioned at the interface with the environment and persistently exposed to airborne pathogens (Bals and Hiemstra, 2004). The recognition of pathogens by lung epithelial cells thus plays an important role in the lung inflammation induced by LPS (Diamond et al., 2000; Bals and Hiemstra, 2004). Toll-like receptor (TLR) 4 was originally thought to be the sole pattern recognition receptor for LPS (Neyen and Lemaitre, 2016), and its activation induces lung inflammation (Chen et al., 2019) or inflammatory responses in lung epithelial cells (Liu et al., 2018). Emerging evidence suggests that transient receptor potential ankyrin 1 (TRPA1) is a non-TLR membrane-bound sensor of LPS (Boonen et al., 2018a; Mazgaeen and Gurung, 2020). TRPA1 is a type of non-selective transmembrane cation channel that is involved in Ca^2+^ permeability (Fernandes et al., 2012). TRPA1 is predominately expressed in primary sensory neurons but also expressed in various non-neuronal cell types (Fernandes et al., 2012; Nassini et al., 2012; Tian et al., 2020), including lung epithelial cells (Nassini et al., 2012; Büch et al., 2013; Lin et al., 2015; Prandini et al., 2016). LPS can directly activate TRPA1 in mouse sensory neurons (Meseguer et al., 2014; Boonen et al., 2018b; Startek et al., 2018) and in human HEK293T cells transfected with TRPA1 (Soldano et al., 2016). However, studies on the functional importance of TRPA1 in LPS-induced consequences have reported equivocal findings. TRPA1 protects against LPS-induced pneumonitis (Hajna et al., 2020) and does not contribute to LPS-induced bladder inflammation (Kamei et al., 2018) in mice. However, TRPA1 mediates LPS-induced lung inflammation in mice (Liu et al., 2020) or inflammatory responses to LPS *in vitro* (Prandini et al., 2016; Yin et al., 2018). Thus, the role of TRPA1 in LPS-induced inflammation has not been conclusively defined.

LPS elevates the intracellular levels of NADPH oxidase-derived reactive oxygen species (ROS), which trigger inflammatory responses in lung epithelial cells (Cho et al., 2016; Dong and Yuan, 2018). NADPH oxidase is the primary enzyme system that generates ROS in mammalian cells (Bedard and Krause, 2007). LPS also induces lung inflammation via mitogen-activated protein kinase/nuclear factor-κB (MAPK/NF-κB) signaling (Cho et al., 2016; Jiang et al., 2016; Chen et al., 2019), which is sensitive to ROS (Mossman et al., 2006; Rahman and Adcock, 2006; Lin et al., 2017). We recently reported (Lin et al., 2015) that cigarette smoke can activate TRPA1 leading to an increase in Ca^2+^ influx in lung epithelial cells. The increased intracellular Ca^2+^ contributes to the activation of NADPH oxidase and results in increased intracellular ROS, which activates the MAPK/NF-κB signaling and leads to interleukin 8 (IL-8) induction (Lin et al., 2015). However, the underlying cellular mechanisms were not investigated in studies demonstrating the contribution of TRPA1 in LPS-induced inflammation (Prandini et al., 2016; Yin et al., 2018; Liu et al., 2020) and are yet to be determined.

The objectives of this study were to investigate the cellular mechanisms of the inflammatory role of TRPA1 in LPS-induced IL-8 responses using primary human bronchial epithelial cells (HBECs) with LPS exposure, and to determine the contribution of TRPA1 to LPS-induced lung inflammation using a murine model (Ueda et al., 2008).

## Materials and Methods

### Drugs and Reagents

Antibodies (Abs) and enzyme-linked immunosorbent assay (ELISA) kits for the detection of IL-8, IL-1β, IL-6, and macrophage inflammatory protein 2 (MIP-2) were purchased from R&D Systems (Minneapolis, MN, United States). Rabbit Ab against c-Jun N-terminal kinase (JNK) was obtained from Cell Signaling (Beverly, MA, United States). Mouse Ab against phospho-JNK was purchased from BD (San Jose, CA, United States). The Screen Quest Fluo-8 Medium Removal Calcium Assay Kit was obtained from AAT Bioquest (Sunnyvale, CA, United States). Membrane-permeable probes hydroethidine (HE) and 2’,7’-dichlorofluorescin diacetate were obtained from Molecular Probes (Eugene, OR, United States). The EnzyChrom NADP^+^/NADPH Assay Kit was purchased from BioAssay Systems (Hayward, CA, United States). Abs against extracellular signal-regulated kinase (ERK), phospho-ERK, and p65 were obtained from Santa Cruz Biotechnology (Santa Cruz, CA, United States). *Escherichia coli* LPS and mouse Abs against α-tubulin, HC030031, ethylene glycol tetraacetic acid (EGTA), N-acetyl-cysteine (NAC), apocynin, and TAK-242 were purchased from Sigma-Aldrich (St. Louis, MO, United States). Mouse Ab against TRPA1 was obtained from Calbiochem (San Diego, CA, United States). Mouse Ab against histone H1 was purchased from Millipore (Bedford, MA, United States). Mouse Ab against 4-hydroxynonenal (4-HNE) was purchased from Abcam (Cambridge, MA, United States). Scramble and TRPA1 small interfering RNAs (siRNAs) were purchased from Ambion (Austin, TX, United States). INTERFERin siRNA transfection reagent was obtained from Polyplus (New York, NY, United States). More information for LPS and antibodies used in this study are provided in Supplementary Table S1.

### Cell Culture

HBECs (Cascade Biologics, Portland, OR, United States) were cultured in epithelial cell growth medium (medium 200; Cascade Biologics, United States) at 37°C in an incubator with 5% CO_2_ with a method described previously (Lin et al., 2015).

### Assessment of Levels of Intracellular Ca^2+^

Intracellular Ca^2+^ levels were measured using Screen Quest^TM^ Fluo-8 Medium Removal Calcium Assay Kit based on the instructions from the manufacturer.

### Measurement of Levels of Intracellular ROS

Membrane-permeable HE was employed to assess the levels of ROS using the methods described previously (Liu et al., 2005); red fluorescent ethidium (ETH) is formed when HE is oxidized by ROS (Benov et al., 1998). HBECs were incubated in culture medium containing 10 μM HE at 37°C for 30 min. The cells were stimulated with LPS for the desired time and then washed and detached with trypsin/EDTA for the measurement of intracellular ROS levels. The fluorescence intensities of the culture medium and cells were analyzed using a multilabel counter (PerkinElmer, Waltham, MA, United States) at 530 nm excitation and 620 nm emission for ETH. Images of the cells were obtained using a Nikon TE2000-U florescence microscope (Tokyo, Japan).

### Determination of NADPH Oxidase Activity

The activity of NADPH oxidase was examined using an EnzyChrom^TM^ NADP^+^/NADPH Assay Kit according to the manufacturer’s instructions. The assay kit measures the change in NADP^+^/NADPH ratio in the samples of cellular lysates to reflect the relative NADPH oxidase activity.

### Transfection of siRNA in HBECs

HBECs were transfected with scramble or TRPA1 siRNA using INTERFERin siRNA transfection reagent for 24 h. The information of the TRPA1 siRNA was reported previously (Lin et al., 2015).

### Mouse Model of LPS-Induced Lung Inflammation

All animal experiments were approved by the Institutional Animal Care and Use Committee of Taipei Veterans General Hospital (Approval Number: IACUC 2017-266). Briefly, 8 week old male *trpa1*^–/–^ mice (Jackson Laboratory, Maine, United States) and wild-type C57BL/6J mice (National Laboratory Animal Center, Taipei, Taiwan) were randomly assigned to four study groups. Either *E. coli* LPS (20 mg/kg) or PBS (the vehicle) was administered via intraperitoneal injection (Ueda et al., 2008). The groups were PBS-wild-type, LPS-wild-type, PBS-*trpa1*^–/–^, and LPS-*trpa1*^–/–^. At the end of each experiment after 24 h of exposure to PBS or LPS, the mice were euthanized using CO_2_.

### Bronchoalveolar Lavage Fluid (BALF) and Lung Tissues

The method of preparation of BALF in the right lung has been described previously (Lin et al., 2015). The BALF samples were centrifuged at 350 × *g* for 5 min at 4°C, and the supernatant of the first lavage fluid was stored at -80°C for the analysis of total protein using a Bio-Rad protein assay reagent (Bio-Rad Laboratories, Hercules, CA, United States). The cell pellets of the BALF samples were re-suspended in PBS for cell counting. Furthermore, the right lung was stored at -80°C for subsequent analysis.

### Histological Assessments

The left lung was fixed with 4% paraformaldehyde and embedded in paraffin. The tissue blocks were cut into 8 μm sections. The sections were deparaffinized, rehydrated, stained with hematoxylin and eosin (H&E), and viewed under a microscope (Motic TYPE 102M, Xiamen, China). A pathologist who was blinded to the data performed the histological assessments based on a method described previously (Yu et al., 2012). The assessment was scored on a scale of 0 (normal) to 5 (maximal). The degree of leukocyte infiltration in peribronchiolar areas was judged by the number of infiltrated leucocytes. The sum of the score of cell infiltration and damage levels, including the thickening of the alveolar walls and epithelium yielded the lung inflammatory score.

### Western Blot Analysis

Aliquots of cell lysates or tissue lysates were separated on 8–12% sodium dodecyl sulfate–polyacrylamide gel electrophoresis and then transblotted onto Immobilon^TM^-P membrane (Millipore). The blots were blocked and incubated with various primary antibodies and the appropriate secondary antibodies. Specific protein bands were detected using an enhanced chemiluminescence kit (PerkinElmer). Quantifications of band densities were performed using the ImageQuant 5.2 software (Healthcare Bio-Sciences, Philadelphia, PA, United States).

### Measurement of 4-Hydroxynonenal (4-HNE) Level

The levels of 4-HNE-modified proteins in lung tissues were measured by Western blot analysis to serve as an index of oxidative stress (Connor et al., 2012).

### Measurements of IL-8, IL-1β, IL-6, and MIP-2 Concentrations

The appropriate ELISA kits were employed to determine the concentrations of IL-8, IL-1β, and IL-6 in culture medium and the concentrations of MIP-2 in BALF and lung tissue.

### Statistical Analysis

The results are presented as mean ± standard error of mean (SEM). Statistical evaluations involved one-way ANOVA followed by Dunnett’s test or Fisher’s least significant difference for multiple comparisons as appropriate. Differences were considered statistically significant at *P* < 0.05.

## Results

### LPS Increases the Production of Inflammatory Cytokines in HBECs

**FIGURE 1:**
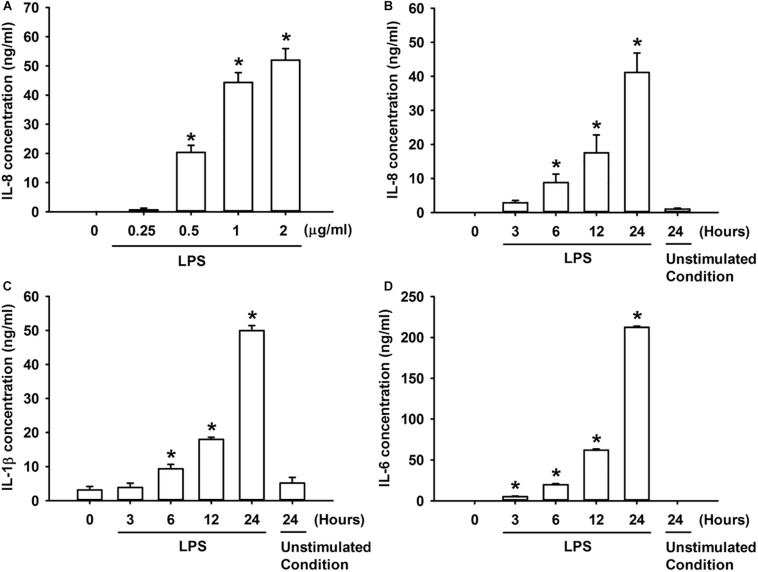
LPS increases the production of IL-8 **(A,B)**, IL-1β **(C)**, and IL-6 **(D)** in HBECs. **(A)** Cells were exposed to 0–2 μg/ml LPS for 24 h. **(B–D)** Cells were exposed to medium alone at time 0 for 24 h (unstimulated condition) or to 1 μg/ml LPS for the indicated times. Protein levels of IL-8 **(A,B)**, IL-1β **(C)**, and IL-6 **(D)** in the culture medium were determined by ELISA. Data in each group are mean ± SEM from four independent experiments. *P < 0.05 vs. time 0.

We first studied the effect of LPS on the production of IL-8 in HBECs. The exposure of HBECs to various concentrations (0–2 μg/ml) of LPS for 24 h resulted in a concentration-dependent increase in IL-8 levels in the cell medium (Figure 1A). Furthermore, the exposure of HBECs to 1 μg/ml LPS for up to 24 h increased the IL-8 levels in cell medium (Figure 1B) and increased the release of IL-1β and IL-6 from HBECs in a time-dependent manner (Figures 1C,D). Thus, 1 μg/ml LPS was selected as the standard challenge for subsequent studies.

### TRPA1 Mediates LPS-Induced IL-8 Expression in HBECs

**FIGURE 2:**
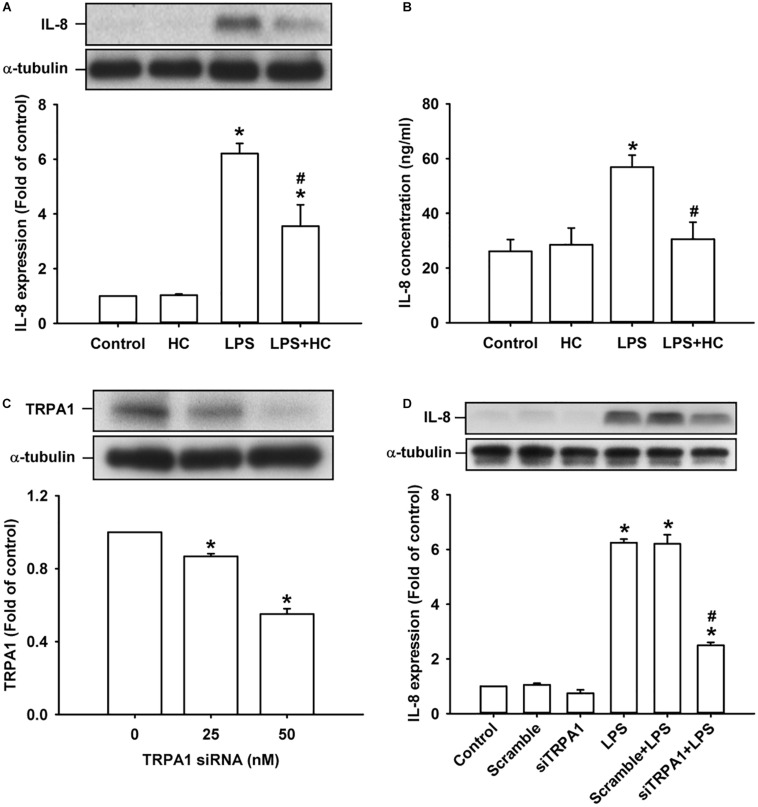
The LPS-induced IL-8 expression was alleviated by treatment with a TRPA1 antagonist or TRPA1 siRNA in HBECs. **(A,B)** Cells were exposed to medium alone or 1 μg/ml LPS for 24 h with or without pretreatment of HC030031 (HC, a TRPA1 antagonist, 9 μM). **(C)** Cells were treated with different concentrations of TRPA1 siRNA (0, 25, and 50 nM). **(D)** Cells were incubated with medium alone or 1 μg/ml LPS for 24 h with pretreatment of 50 nM siRNA (siTRPA1) or scramble siRNA. The protein levels in cell lysate **(A,C,D)** and cell medium **(B)** were measured using Western blot and ELISA, respectively. Data in each group are mean ± SEM from four independent experiments. *P < 0.05 vs. the control; ^#^*P* < 0.05 vs. LPS alone.

We then explored the role of TRPA1 in the inflammatory responses of HBECs to LPS. The exposure of HBECs to LPS for 24 h increased the intracellular (Figure 2A) and extracellular expression of IL-8 (Figure 2B), which were largely or nearly prevented by pretreatment with the TRPA1 antagonist, HC030031 (9 μM); this dose was then adapted from our recent study (Lin et al., 2015). Additionally, pretreatment with TRPA1 siRNA (0, 25, and 50 nM) effectively reduced the expression of TRPA1 protein in HBECs (Figure 2C). Importantly, the induction of IL-8 by LPS was substantially attenuated by pretreatment with TRPA1 siRNA (50 nM), but was unaffected by pretreatment with scramble siRNA (Figure 2D).

### TRPA1 Mediates LPS-Induced Increase in Intracellular ROS via the Ca^2+^-Dependent Activation of NADPH Oxidase in HBECs

**FIGURE 3:**
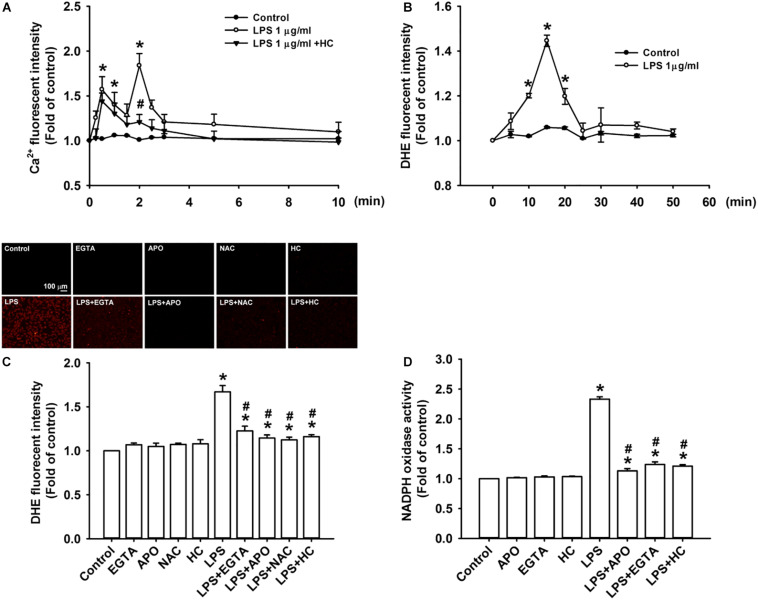
TRPA1 mediates LPS-induced increase in intracellular ROS via the Ca^2+^-dependent activation of NADPH oxidase in HBECs. **(A)** Cells were exposed to medium alone, 1 μg/ml LPS, or the combination of LPS and HC030031 (HC, 9 μM) for 0, 10, 30, 60, 90, and 120 s and 3, 5, and 10 min. **(B)** Cells were treated with or without LPS (1 μg/ml) for indicated times. **(C)** Cells were exposed to medium alone or 1 μg/ml LPS for 15 min after pretreatment with EGTA (500 μM), apocynin (APO, 150 μM), NAC (1 mM), or HC (9 μM). HC, EGTA, APO, and NAC are a TRPA1 antagonist, an extracellular Ca^2+^ chelator, an inhibitor of NADPH oxidase, and a ROS scavenger, respectively. **(D)** Cells were exposed to medium alone or 1 μg/ml LPS for 10 min after pretreatment with EGTA (500 μM), APO (150 μM), or HC (9 μM). Intracellular levels of Ca^2+^ **(A)** and ROS **(B,C)** and NADPH oxidase activity **(D)** were measured by Fluo-8, HE/DTH, and NADP^+^/NADPH fluorescent probe assays, respectively. Data in each group are mean ± SEM from four independent experiments. *P < 0.05 vs. the control; ^#^*P* < 0.05 vs. LPS alone.

We further studied the role of TRPA1 in evoking the early responses of HBECs to LPS. The exposure of HBECs to LPS immediately produced two peak increases in intracellular Ca^2+^ level at 30 s and 2 min after treatment initiation; this increase rapidly declined 3 min after treatment initiation (Figure 3A). The LPS-induced increase in intracellular Ca^2+^ level measured at 2 min was inhibited by pretreatment with TRPA1 antagonist, HC030031. The intracellular ROS level in HBEC was substantially increased 10 min after exposure to LPS and reached the peak 15 min after treatment initiation (Figure 3B). The LPS-induced increase in intracellular ROS was remarkably attenuated by pretreatment with EGTA (an extracellular Ca^2+^ chelator), apocynin (an inhibitor of NADPH oxidase), NAC (a ROS scavenger), or HC030031 (Figure 3C). Further analysis revealed that LPS considerably increased the activity of NADPH oxidase at 10 min after exposure, and this effect was markedly inhibited by pretreatment with EGTA, apocynin, or HC030031 (Figure 3D).

### TRPA1 Mediates the LPS-Induced Activation of ERK/JNK/NF-κB Signaling in HBECs via the Functions of Intracellular Ca^2+^ and ROS

**FIGURE 4:**
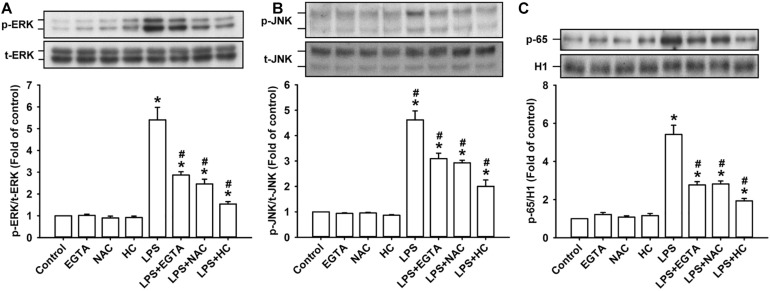
TRPA, intracellular Ca^2+^, and intracellular ROS contribute to the LPS-induced activation of the ERK/JNK/NF-κB signaling in HBECs. Cells were exposed to medium alone or 1 μg/ml LPS for 3 **(A,B)** and 9 h **(C)** and pretreated with EGTA (an extracellular Ca^2+^ chelator, 500 μM), NAC (ROS scavenger, 1 mM), or HC030031 (HC, a TRPA1 antagonist, 9 μM). Protein expression was analyzed by Western blot. The increased phosphorylation of kinases indicates the activation of ERK and JNK. The increased presence of the p65 subunit in the nucleus indicates the activation of NF-κB. Data in each group are mean ± SEM from four independent experiments. *P < 0.05 vs. the control; ^#^*P* < 0.05 vs. LPS alone.

The activation of ERK, JNK, and NF-κB is known to be a signaling pathway that is central to the induction of IL-8 in HBECs (Lin et al., 2015, 2017). ERK and JNK are two MAPK subfamilies (Mossman et al., 2006). We found that the exposure of HBECs to LPS for 3 h increased the levels of phosphorylated ERK (Figure 4A) and phosphorylated JNK (Figure 4B). The incubation of HBECs with LPS for 9 h increased the expression of the p65 subunit of NF-κB in the nucleus (Figure 4C). This activation of MAPK/NF-κB signaling by LPS was greatly reduced by pretreatment with EGTA, NAC, or HC030031.

### The Contribution of TRPA1 to LPS-Induced Responses in HBECs Is Independent of TLR4

**FIGURE 5:**
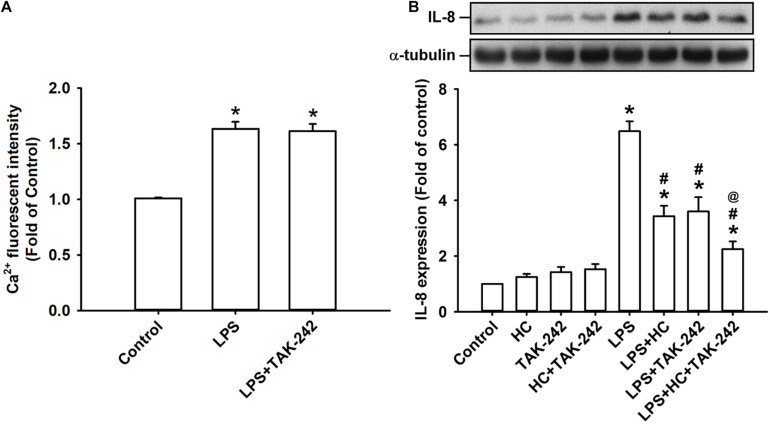
Both TRPA1 and TLR4 contribute to LPS-induced IL-8 expression. **(A)** Cells were exposed to medium alone, 1 μg/ml LPS for 24 h, or 1 μg/ml LPS with pretreatment with TAK-242 (a TLR4 antagonist, 1 μM). Intracellular levels of Ca^2 +^ were measured by Fluo-8 fluorescent probe assays. **(B)** Cells were exposed to medium alone or 1 μg/ml LPS for 24 h and pretreated with HC030031 (HC, a TRPA1 antagonist, 9 mM), TAK-242 (a TLR4 antagonist, 1 μM), or the combination of HC and TAK-242. The protein levels in cell lysate were measured using Western blot. Data in each group are mean ± SEM from four independent experiments. *P < 0.05 versus the control; ^#^*P* < 0.05 versus LPS alone; ^@^*P* < 0.05 versus LPS + HC or LPS + TAK-242.

The activation of TLR4 by LPS induces inflammatory responses in lung epithelial cells (Chen et al., 2019). We found that the exposure of HBECs to LPS for 2 min caused an increase in intracellular Ca^2+^ level, and this increase was unaffected by pretreatment with TAK-242 (a TLR4 inhibitor) (Matsunaga et al., 2011) as shown in Figure 5A. Also, the LPS-induced IL-8 production measured at 24 h after exposure in HBECs was substantially attenuated by pretreatment with HC030031 or TAK-242 alone (Figure 5B). Notably, the LPS-induced IL-8 production was further reduced by pretreatment with the combination of HC030031 and TAK-242 (Figure 5B).

### LPS-Induced Inflammation and Oxidative Stress in Lung Tissues Are Lessened in *trpa1^–/–^* Mice

**FIGURE 6:**
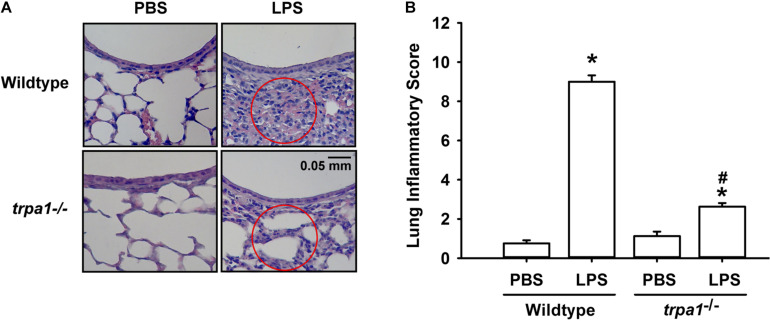
LPS-induced lung inflammation and lung inflammatory scores are reduced in *trpa1^– /–^* mice. Mice were treated with an intraperitoneal injection of *E. coli* LPS (20 mg/kg) or PBS (the vehicle). **(A)** Representative images of H&E-stained lung sections obtained from PBS- or LPS-exposed wild-type and *trpa1^– /–^* mice. Circles indicate the areas of inflammatory cell infiltration. **(B)** Lung inflammatory scores were calculated according to the sum of the levels of cell infiltration and damage levels assessed from lung sections. Data in each group are mean ± SEM from eight mice. *P < 0.05 vs. the PBS exposure group in both genotypes; ^#^*P* < 0.05 vs. the LPS-exposed wild-type group.

**FIGURE 7:**
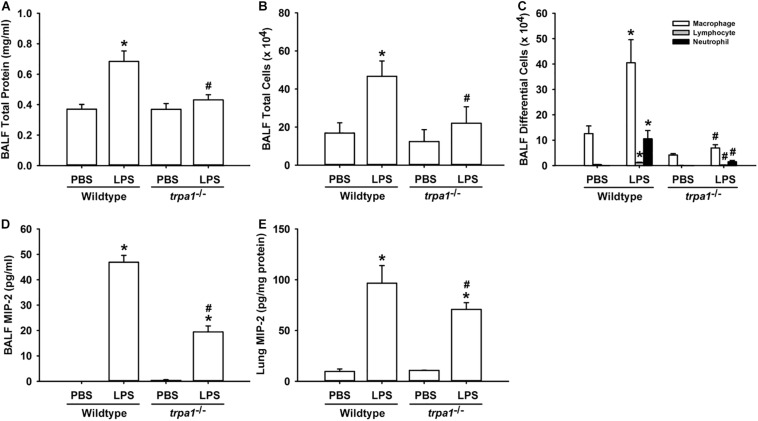
LPS-induced inflammatory responses in the lungs are alleviated in *trpa1^– /–^* mice. Mice were treated with an intraperitoneal injection of *E. coli* LPS (20 mg/kg) or PBS (the vehicle). Total protein contents **(A)**, total cell count **(B)**, and differential cell count **(C)** in the BALF at 24 h after PBS or LPS treatment as indications of lung inflammation. Levels of MIP-2 in BALF **(D)** and lung tissues **(E)** were analyzed by ELISA. Data in each group are mean ± SEM from eight mice. *P < 0.05 vs. the PBS-exposed group in both genotypes; ^#^*P* < 0.05 vs. the LPS-exposed wild-type group.

**FIGURE 8:**
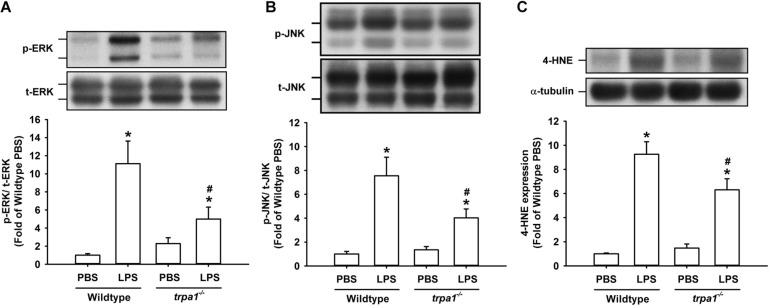
LPS-induced ERK/JNK activation and oxidative stress in lung tissues are reduced in *trpa1^– /–^* mice. Mice received an intraperitoneal injection of *E. coli* LPS (20 mg/kg) or PBS (the vehicle). **(A,B)** The increased phosphorylation of kinases indicates the activation of ERK and JNK. **(C)** LPS-induced increase in oxidative stress was indicated by increased 4-HNE expression. Protein expression was analyzed by Western blot. Data in each group are mean ± SEM from eight mice. *P < 0.05 vs. the PBS-exposed group in both genotypes; ^#^*P* < 0.05 vs. the LPS-exposed wild-type group.

The histological lung section of LPS-exposed wild-type mice displayed an extensive infiltration of inflammatory cells, thickening of the alveolar walls, and the presence of abnormal re-epithelialization (Figure 6A). These pathohistological changes were found to be lesser in the LPS-exposed *trpa1^–/–^* mice (Figure 6A). Comparisons of the group data of lung inflammatory scores confirmed the difference in the degree of lung inflammation between the LPS-exposed wild-type and *trpa1^–/–^* mice (Figure 6B). Furthermore, as compared to the PBS-exposed wild-type mice, LPS-exposed wild-type mice exhibited increases in total protein levels (Figure 7A), total cell counts (Figure 7B), differential cell counts (Figure 7C), and MIP-2 levels (Figure 7D) in BALF, and increases in MIP-2 levels (Figure 7E) in lung tissue samples. LPS-exposed wild-type mice also displayed increased levels of phosphorylated ERK (Figure 8A), phosphorylated JNK (Figure 8B), and 4-HNE (Figure 8C) in lung tissue samples. These indices of inflammation, the activation of MAPK signaling, and oxidative stress were remarkably lower in *trpa1^–/–^* mice exposed to LPS (Figures 6–8).

## Discussion

Our *in vitro* studies showed that LPS increased the production of several inflammatory mediators, including IL-8, IL-1β, and IL-6, in HBECs (Figure 1). All these inflammatory mediators are important for the induction of LPS-induced lung inflammation (Tsuruta et al., 2007; Planagumà et al., 2015). We then chose IL-8 as the target response to study the contribution of TRPA1 and its underlying cellular mechanisms. The contribution of TRPA1 as evidenced by our finding is that TRPA1 antagonist or TRPA1 siRNA substantially reduced the LPS-induced IL-8 (Figure 2). Mechanistically, we demonstrated that LPS sequentially elevated Ca^2+^ level, increased ROS level, and activated MAPK/NF-κB signaling in HBECs. We identified the cascade of these cellular events using various experimental interventions. Initially, exposure to LPS activated the TRPA1 of HBECs resulting in an increase in Ca^2+^ influx (Figure 3). This increase in intracellular Ca^2+^ then promoted the NADPH oxidase activation, which led to the elevation of the intracellular ROS level (Figure 3). Finally, the increased ROS activated the MAPK/NF-κB signaling pathway (Figure 4), which resulted in the IL-8 induction. We further showed that the contribution of TRPA1 is at least in part independent of TRL4 using a TLR4 antagonist as the intervention (Figure 5). These observations suggest that the activation of lung epithelial TRPA1 by LPS contributes to the induction of IL-8 via a Ca^2+^-dependent signaling pathway in HBECs. This inflammatory signaling is similar to that reported by our recent study when cigarette smoke was used as the stimulus to activate lung epithelial TRPA1 (Lin et al., 2015).

NADPH oxidase-derived ROS (Cho et al., 2016; Dong and Yuan, 2018) and MAPK/NF-κB signaling (Cho et al., 2016; Jiang et al., 2016; Chen et al., 2019) are involved in LPS-induced lung inflammation. In this study, the LPS-induced increase in intracellular Ca^2+^ appears to be mediated though TRPA1. This increase in intracellular Ca^2+^ may serve as an important trigger to activate an inflammatory signaling for the induction of IL-8. We found that this Ca^2+^ signaling is essential for the activation of NADPH oxidase, which leads to an excess production of intracellular ROS. Indeed, intracellular Ca^2+^ has been shown to be an upstream signal for the NADPH oxidase activation during cellular stress (Trebak et al., 2010; Jiang et al., 2011). Our finding that intracellular ROS subsequently activated MAPK/NF-κB signaling is not surprising, because this pathway is ROS-sensitive (Mossman et al., 2006; Rahman and Adcock, 2006; Lin et al., 2017). We, however, cannot exclude the possibility that the TRPA1-mediated increase in intracellular Ca^2+^ also serves as a Ca^2+^ signal to activate downstream inflammatory signaling. For example, an increased intracellular Ca^2+^ can activate the MAPK/NF-κB pathway to induce IL-8 in lung epithelial cells (Carmona et al., 2010). Thus, lung epithelial TRPA1 may detect the presence of LPS and transduce this information into the transcriptional regulation of lung inflammation via a Ca^2+^-dependent signaling pathway. Our notion is in line with the current concept that TRPA1 is a non-TLR membrane-bound sensor of LPS (Boonen et al., 2018a; Mazgaeen and Gurung, 2020). This concept is supported by increasing evidence that LPS can directly activate TRPA1 (Meseguer et al., 2014; Soldano et al., 2016; Boonen et al., 2018b) via a mechanism that involves the mechanical perturbations of the plasma membrane (Boonen et al., 2018a; Startek et al., 2018; Mazgaeen and Gurung, 2020). In fact, TRPA1 is not the only type of TRP channel that can detect the presence of LPS (Boonen et al., 2018a; Mazgaeen and Gurung, 2020). For example, LPS also activates transient receptor potential vanilloid 4 (TRPV4) within seconds in lung epithelial cells and produce protective responses, including direct antimicrobial action and increase in airway clearance (Alpizar et al., 2017). In our study, we found that LPS produced two peak increases in intracellular Ca^2+^ level at 30 s and 2 min after exposure and the first peak increase was unaffected by pretreatment with TRPA1 antagonist (Figure 3A). Perhaps, the activation of other type(s) of TRP channels by LPS is responsible for the first peak increase in Ca^2+^. Collectively, the present study further characterized the LPS-triggered inflammatory signaling in lung epithelial cells and showed that the process depends on having a functional TRPA1 and an influx of Ca^2+^. This study provides additional evidence for the understanding of the early events involved in the induction of IL-8 by LPS in HBECs.

In this study, we further demonstrated that *trpa1^–/–^* mice displayed a lower level of LPS-induced lung inflammation compared with wild-type mice. This observation was based on our findings regarding alleviations of increases in vascular permeability, inflammatory cell infiltration, inflammatory cytokine levels, oxidative stress, and the activation of inflammatory MAPK signaling (Figures 6–8). In our previous study, we have shown that TRPA1 is expressed in the lung epithelium of wild-type mice as assessed by *en face* immunostaining (Lin et al., 2015). Other investigators have suggested that lung epithelium plays an important role in the initiation and progression of LPS-induced lung inflammation (Diamond et al., 2000; Bals and Hiemstra, 2004). Therefore, a lack of epithelial TRPA1 in the *trpa1^–/–^* mice may be at least in part responsible for the reduced lung inflammation observed in our *trpa1^–/–^* mice. TRPA1 is expressed in other types of lung cells, such as smooth muscle cells, fibroblasts, and macrophages (Nassini et al., 2012; Tian et al., 2020), all of which may participate in the development of LPS-induced lung inflammation. Accordingly, the lack of TRPA1 in these lung cells may also contribute to the reduction in lung inflammation that was observed in our *trpa1^–/–^* mice.

Previous findings regarding the role of TRPA1 in LPS-induced inflammation have been equivocal. By comparing the responses between wild-type and *trpa1^–/–^* mice, Hajna et al. (2020) demonstrated that TRPA1 protects against LPS-induced acute pneumonitis and hyperresponsiveness. Kamei et al. (2018), however, reported that TRPA1 plays no role, because TRPA1 knockout does not alter LPS-induced bladder inflammation in mice. Other studies showed that the pharmacological inhibition or gene silencing of TRPA1 alleviates LPS-induced lung inflammation in mice (Liu et al., 2020) or inflammatory responses in lung epithelial cells from patients with cystic fibrosis (Prandini et al., 2016) and in human osteoarthritic fibroblast-like synoviocytes (Yin et al., 2018), suggesting the inflammatory role of TRPA1. Our *in vivo* and *in vitro* studies provide strong evidence in favor of the contribution of TRPA1 to LPS-induced lung inflammation.

Our study had at least two limitations that warrant discussion. Firstly, instead of using a TRPA1 antagonist to treat wild-type mice, we used trpa1*^–/–^* mice as the model to delineate the contribution of TRPA1 to LPS-induced lung inflammation. Secondly, instead of intratracheal or intranasal installation LPS, our animals received intraperitoneal injection of LPS. Whether our findings can be confirmed by using a TRPA1 antagonist or intratracheal/intranasal installation of LPS requires further investigations.

In summary, our *in vitro* findings suggest that exposure to LPS activates lung epithelial TRPA1. This activation sequentially produces a cascade of related cellular events, including the elevation of Ca^2+^ level, increase in ROS level, the activation of MAPK/NF-κB signaling, and the induction of IL-8 in HBECs. Our *in vivo* findings indicate that TRPA1 knockout alleviates LPS-induced inflammation, oxidative stress, and the activation of MAPKs in the lungs of mice. Our findings offer novel information regarding the pathogenic mechanisms associated with LPS-induced lung inflammation and may be valuable for the development of potential therapies.

## Data Availability Statement

The raw data supporting the conclusions of this article will be made available by the authors, without undue reservation, to any qualified researcher.

## Ethics Statement

The animal study was reviewed and approved by the Institutional Animal Care and Use Committee of Taipei Veterans General Hospital (Approval Number: IACUC 2017-266).

## Author Contributions

H-KK, A-HL, and D-WP conducted the studies, analyzed, and interpreted the data. H-KK and A-HL wrote the manuscript. YK and T-SL led the project, interpreted the data, and finished the manuscript. All authors contributed to the article and approved the submitted version.

## Conflict of Interest

The authors declare that the research was conducted in the absence of any commercial or financial relationships that could be construed as a potential conflict of interest.
