# Supplementary material for: Lung Epithelial TRPA1 Mediates Lipopolysaccharide-Induced Lung Inflammation in Bronchial Epithelial Cells and Mice
Source: Front Physiol. 2020 Nov 17;11:596314. doi: 10.3389/fphys.2020.596314 (PMC7705107; doi:10.3389/fphys.2020.596314)
Supplement: Supplementary file 1 [file Table_1.pdf]

Article: Lung Epithelial TRPA1 Mediates Lipopolysaccharide-induced Lung Inflammation in Bronchial Epithelial Cells and Mice

Authors: Hsin-Kuo Ko, An-Hsuan Lin, Diahn-Warng Perng, Tzong-Shyuan Lee and Yu Ru Kou

Supplementary Materials

Table S1. Information for the lipopolysaccharides (LPS) and antibodies used in this study.

**Drug or Antibody**	**Company**	**Catalogue No.**
LPS	Sigma	SI-L2630
IL-8	R & D	MAB208
TRPA1	Abcam	Ab68847
p-ERK	Santa Cruz	SC-7383
t-ERK	Santa Cruz	SC-94
p-JNK	BD	612540
t-JNK	Santa Cruz	SC-7345
p-65	Santa Cruz	SC-101748
H-1	Millipore	#05-457
α-tubulin	Sigma	T5168
4-HNE	Abcam	Ab46545
